# Astrocytes and oligodendrocytes in grey and white matter regions of the brain metabolize fatty acids

**DOI:** 10.1038/s41598-017-11103-5

**Published:** 2017-09-07

**Authors:** Kristina Hofmann, Rosalia Rodriguez-Rodriguez, Anne Gaebler, Núria Casals, Anja Scheller, Lars Kuerschner

**Affiliations:** 1 Life and Medical Sciences Institute (LIMES), University of Bonn, Carl-Troll-Straße 31, D-53115 Bonn, Germany; 2 Basic Sciences Department, Faculty of Medicine and Health Sciences, Universitat Internacional de Catalunya, Sant Cugat del Vallès, Barcelona, Spain; 3 Centro de Investigación Biomédica en Red de Fisiopatología de la Obesidad y la Nutrición (CIBEROBN), Instituto de Salud Carlos III, Madrid, Spain; 4 Department of Molecular Physiology, Center for Integrative Physiology and Molecular Medicine (CIPMM), University of Saarland, Kirrberger Straße 100, D-66424 Homburg, Germany

## Abstract

The grey and white matter regions of the mammalian brain consist of both neurons and neuroglial cells. Among the neuroglia, the two macroglia oligodendrocytes and astrocytes are the most abundant cell types. While the major function of oligodendrocytes is the formation of the lipid-rich myelin structure, the heterogeneous group of astrocytes fulfils a multitude of important roles in cerebral development and homeostasis. Brain lipid homeostasis involves the synthesis of a specific cerebral lipidome by local lipid metabolism. In this study we have investigated the fatty acid uptake and lipid biosynthesis in grey and white matter regions of the murine brain. Key findings were: (i) white matter oligodendrocytes and astrocytes take up saturated and unsaturated fatty acids, (ii) different grey matter regions show varying lipid labelling intensities, (iii) the medial habenula, an epithalamic grey matter structure, and the oligodendrocytes and astrocytes therein are targeted by fatty acids, and (iv) in the medial habenula, the neutral lipid containing lipid droplets are found in cells facing the ventricle but undetectable in the habenular parenchyma. Our data indicate a role for oligodendrocytes and astrocytes in local lipid metabolism of white and grey matter regions in the brain.

## Introduction

The lipid pool of the mammalian brain is partially separated from that of the body by the action of barrier structures such as the blood-brain barrier^1–3^. While depending on dietary supply of essential poly-unsaturated fatty acids (PUFAs), the brain is capable of synthesizing its specific lipidome by local lipid metabolism. Generally, the brain features a high lipid content and is enriched in PUFA-bearing lipids. PUFA-containing lipids cross the endothelial blood-brain barrier by dedicated transport systems that more or less select against saturated and mono-unsaturated fatty acids and complex lipids derived from them^1, 4–7^. At the distal side of the barrier the lipids are released and target the different cell types in the brain. Detailed knowledge on the mammalian intra-cerebral lipid transport is lacking, but a brain-specific pool of lipoproteins is involved^8, 9^. These lipoproteins are widely expressed in the brain and their role for local cholesterol transport is well-established^10^. However, the trafficking of other cerebral lipids and the contributions of lipoproteins and monomeric transport here is less clear.

While *de novo* lipid biosynthesis in the brain occurs, the *in vivo* rates in the various cerebral cell types and their contribution are largely unknown. Comparative lipid profiling revealed, that grey matter is enriched in PUFA-containing phospholipids over white matter, whereas the latter shows strongly elevated levels of some sphingolipids like the cerebrosides^11^. Oligodendrocytes are a dominant cell type of white matter. As they produce the lipid-rich myelin, these macroglial cells likely possess a high capacity for *de novo* lipid synthesis. Neurons are an instrumental part of grey matter and possess an elongated shape with a large membrane to volume ratio. These cells show extensive membrane remodelling activity and have a high turnover of energy^12^. As demonstrated for cholesterol, under normal conditions neurons do not engage strongly in cholesterol biosynthesis, but rather receive this lipid like other energy substrates from donating astrocytes, macroglial cells located in both grey and white matter^10^. Hence, for cholesterol the astrocytes possess a high synthesis capacity, but it remains unclear whether astrocytes also produce and provide other lipids.

This study aims to investigate the local fatty acid metabolism and trafficking in grey and white matter of the mouse brain in a comparative manner using the *in situ* system of acute brain slices and *in vivo* approaches. The goal was to (1) determine whether saturated and unsaturated fatty acids are differentially processed by grey and white matter, (2) identify the involved macroglial cells and elucidate their role in lipid processing, and (3) subsequently validate selected findings from the *in situ* system in an *in vivo* setup.

## Results

### Varying lipid uptake in different grey and white matter regions

**Figure 1:**
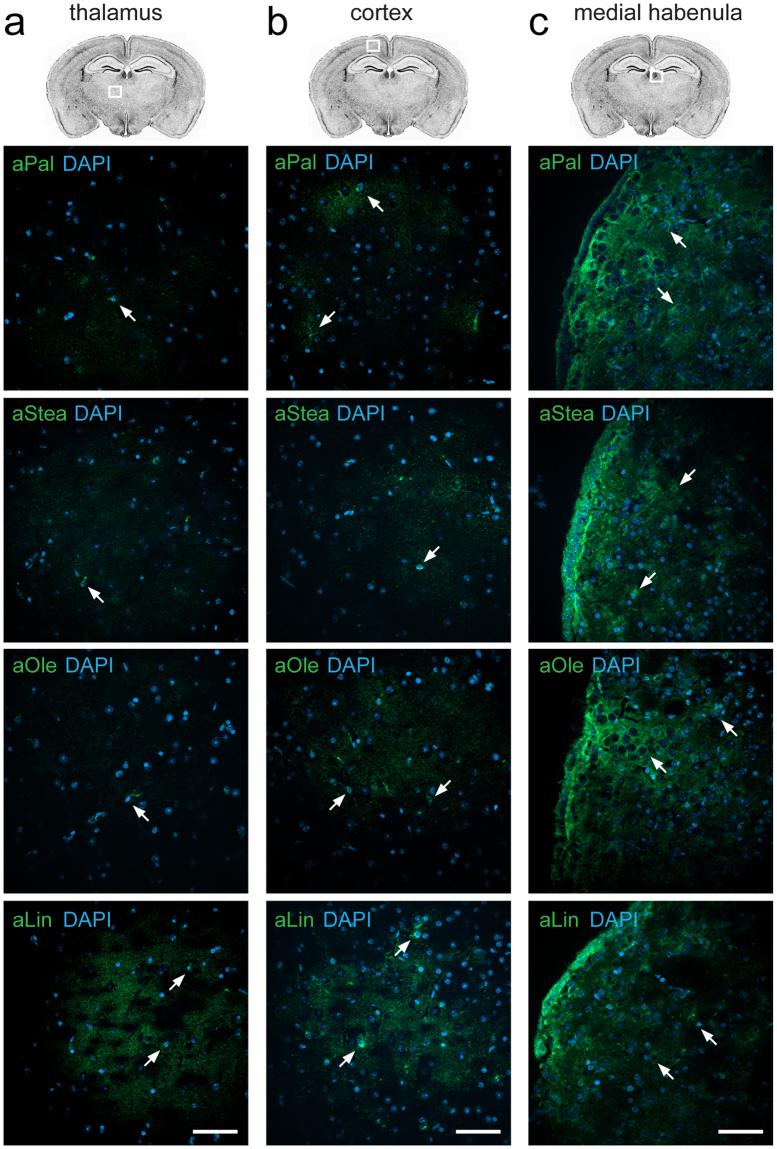
Lipid uptake to grey matter areas. Brain slice cultures of mice were incubated with 50 µM of either the saturated fatty acids alkyne palmitate (aPal) or alkyne stearate (aStea), or the unsaturated fatty acids alkyne oleate (aOle) or alkyne linoleate (aLin) for 2 h. Fluorescence microscopy after click-reaction was performed showing alkyne lipids (green) and nuclei stained by DAPI (blue) in merged channels overview micrographs. Images of the thalamus (**a**) and cortex (**b**) show only weak accumulation of all fatty acids to individual cells (arrows) with the highest signal observed for alkyne linoleate. In the medial habenula (**c**) strong staining by all fatty acids was observed extending from the adjacent ependymal cell layer of the dorsal 3^rd^ ventricle into the parenchyma of the medial habenula. All images were recorded with equal settings and are shown at equal intensity levels. Scale bars, 50 μm.

To determine the fatty acid uptake and lipid distribution in the murine brain traceable alkyne analogues of the abundant fatty acids palmitate, stearate, oleate and linoleate were supplied to acute brain slice cultures. Fluorescence microscopy analysis of various grey matter regions showed only moderate staining in the thalamus and cortex (Fig. 1a,b) and a stronger lipid labelling in the medial habenula (Fig. 1c). In thalamus and cortex the tracer accumulation appeared less pronounced for the saturated palmitate and stearate than for the unsaturated oleate and linoleate (Supplementary Table S1) and for all fatty acids the lipid signal localized to easily discernable individual cells (Fig. 1).

**Figure 2:**
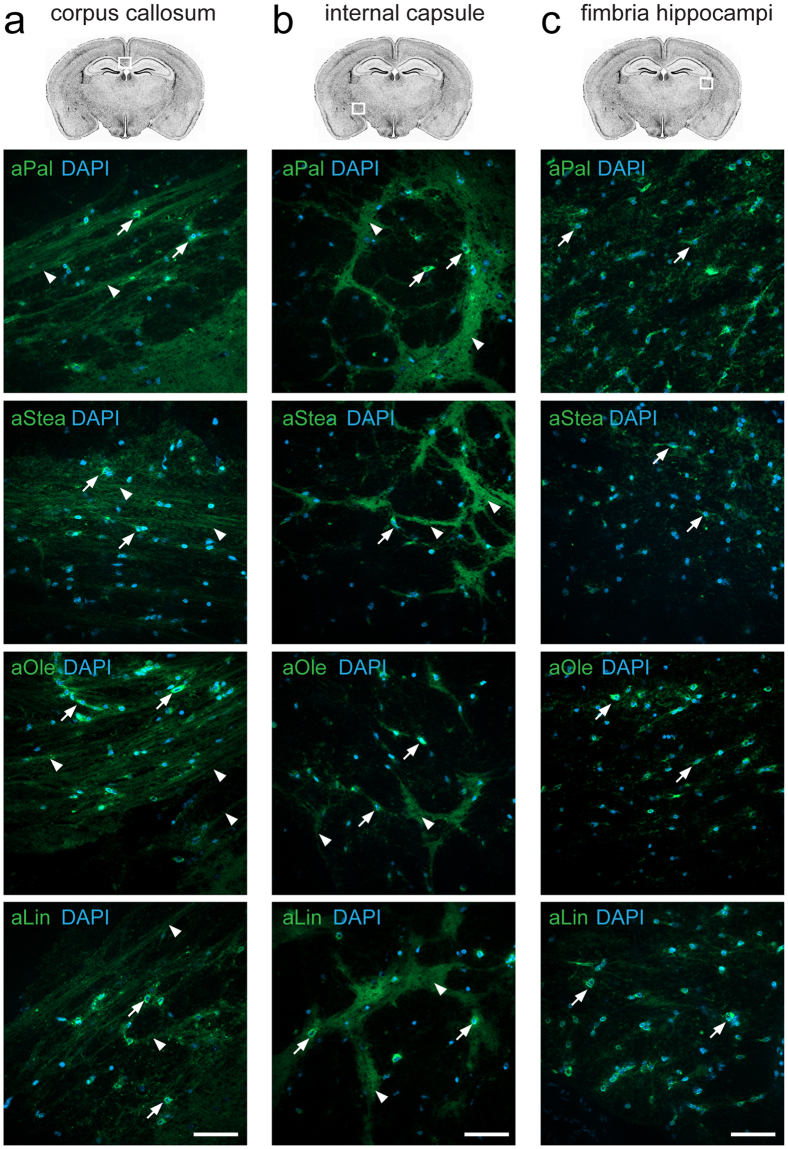
Lipid uptake to white matter areas. Brain slice cultures of mice were incubated with 50 µM of either the saturated fatty acids alkyne palmitate (aPal) or alkyne stearate (aStea), or the unsaturated fatty acids alkyne oleate (aOle) or alkyne linoleate (aLin) for 2 h. Fluorescence microscopy after click-reaction was performed showing alkyne lipids (green) and nuclei stained by DAPI (blue) in merged channels overview micrographs. Images of the corpus callosum (**a**), internal capsule (**b**), and fimbria hippocampi **(c)** show strong accumulation of all fatty acids to individual cells (arrows) and fibres (arrow heads). All images were recorded with equal settings and are shown at equal intensity levels. Scale bars, 50 μm.

Various white matter regions were also analysed (Fig. 2). Here all four fatty acids tested gave a pronounced staining. In the corpus callosum (Fig. 2a) and internal capsule (Fig. 2b) the lipid signal was observed both in defined cellular structures around nuclei and in fibres extending hundreds of micrometres and reminiscent of myelin. In the fimbria hippocampi the lipid staining was largely confined to cell somas, and substantially less abundant in putative myelin structures (Fig. 2c).

The uptake and distribution of alkyne lipids depended on active metabolism as samples incubated in the absence of alkyne lipid (Supplementary Fig. S1) or prefixed before cultivation and lipid incubation (Supplementary Fig. S2) lacked acknowledgeable alkyne lipid signal in both grey and white matter.

### Brain lipid metabolism involves astrocytes and oligodendrocytes

**Figure 3:**
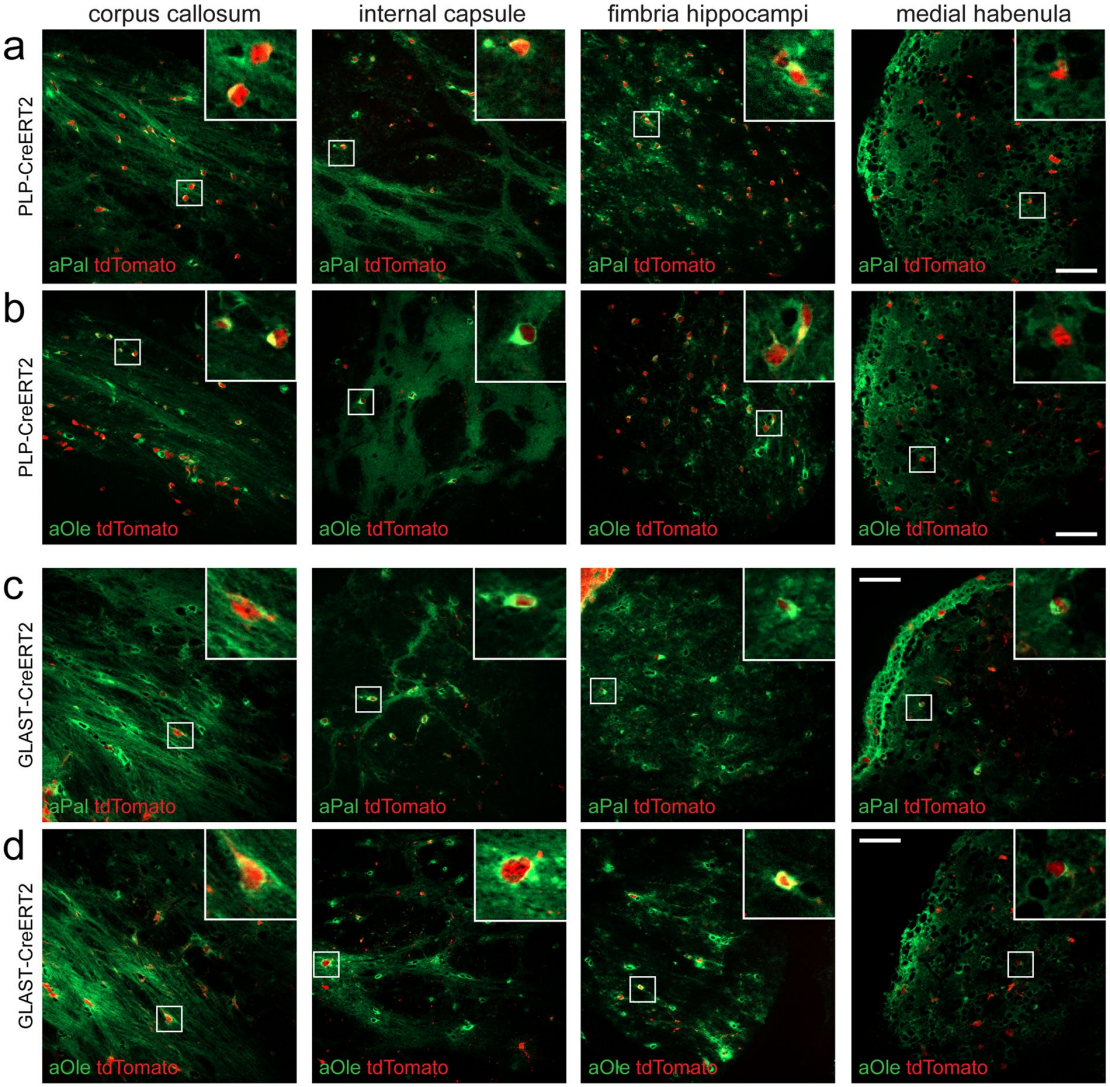
Alkyne lipids localize to reporter-expressing oligodendrocytes and astrocytes. (**a**,**b**) Brain slices of transgenic mice expressing the red-fluorescent tdTomato-reporter after tamoxifen-induced recombination in (**a**,**b**) oligodendrocytes (PLP-CreERT2), or (**c**,**d**) astrocytes (GLAST-CreERT2) were incubated with 50 µM of (**a**,**c**) alkyne palmitate (aPal), or (**b**,**d**) alkyne oleate (aOle) for 2 h. Fluorescence microscopy after click-reaction was performed showing alkyne lipids (green) and fluorescent protein (red) in merged channel overview micrographs. Inserts depict close-up images of individual cells with co-localizing signals. All images were recorded with equal settings and are shown at equal intensity levels. Scale bars, 50 μm.

Some of the cells labelled by the various fatty acid tracers morphologically resembled macroglia i.e. astrocytes and oligodendrocytes. In order to reliably identify these macroglia and confirm their participation in brain lipid metabolism, microscopy co-localization experiments were performed (Fig. 3). For this, transgenic fluorescent reporter mice were used that expressed the fluorescent tdTomato protein after tamoxifen-induced recombination in cells with active promoters for proteolipid protein (PLP), or glutamate aspartate transporter (GLAST) driving CreERT2 expression. While PLP is a well-established marker for mature myelinating oligodendrocytes^13^, GLAST is expressed by many astrocytes^14^. In acute brain slices the tdTomato-expressing oligodendrocytes could be labelled by either alkyne palmitate or alkyne oleate, the main saturated or unsaturated fatty acids, respectively (Fig. 3a,b). Reporter-marked astrocytes were also positive for both lipids (Fig. 3c,d). Taken together, both macroglial cell types occurring in white and grey matter are targeted by saturated and unsaturated fatty acids.

### Lipids both *in situ* and *in vivo* target the medial habenula

**Figure 4:**
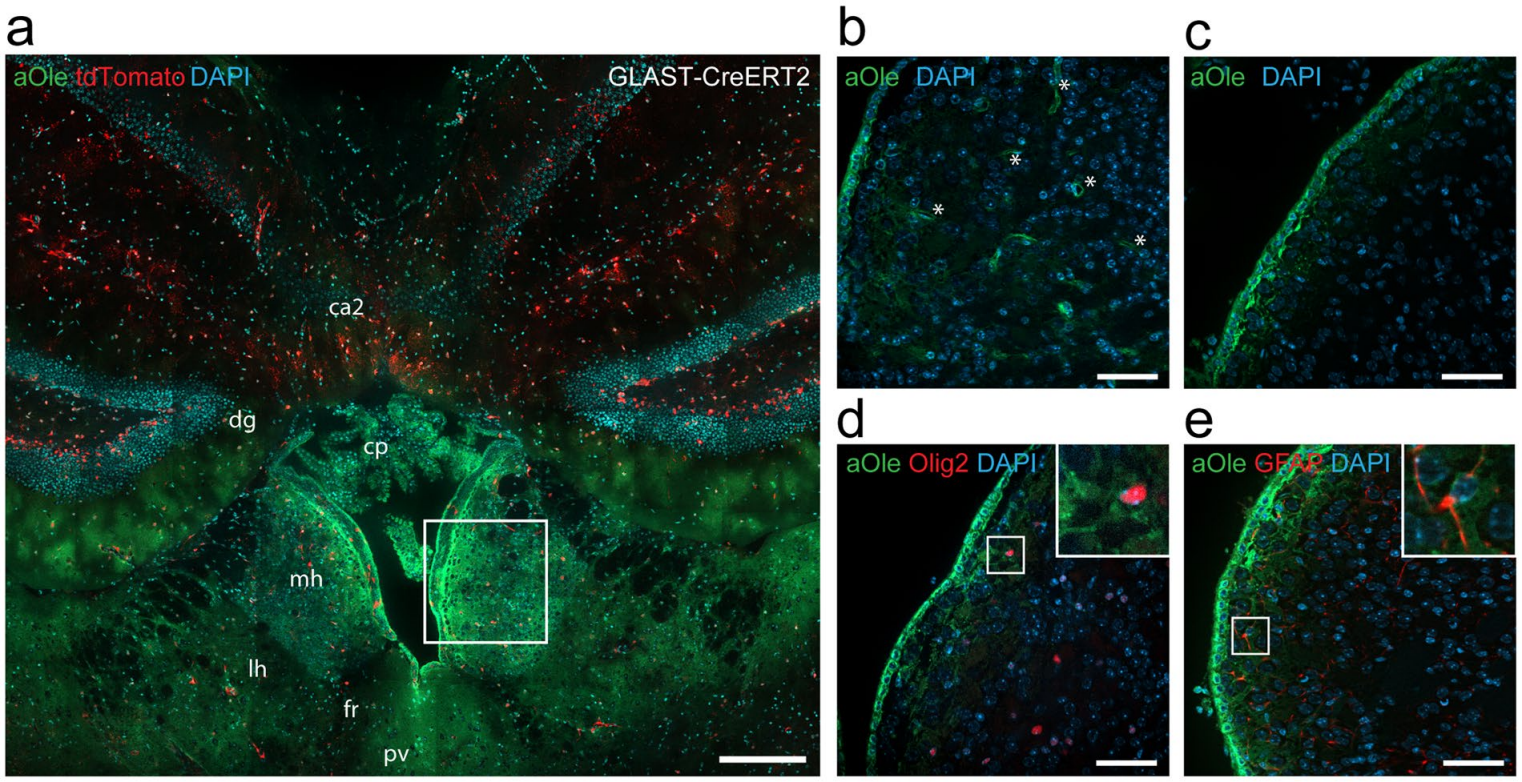
The alkyne lipid uptake to the medial habenula *in vivo* corresponds to the *in situ* observations. (**a**) *In situ* cultures of brain slices from of transgenic mice expressing fluorescent tdTomato-reporter after tamoxifen-induced recombination in astrocytes (GLAST-CreERT2) were incubated with 50 µM of alkyne oleate (aOle) for 2 h. Fluorescence microscopy after click-reaction was performed showing alkyne lipids (green), fluorescent protein (red) and nuclei stained by DAPI (blue) in merged channel micrographs. Overview images show medial habenula (mh), lateral habenula (lh), fasciculus retroflexus (fr), paraventricular thalamic nucleus (pv), dentate gyrus (dg), field CA2 of hippocampus (ca2) and choroid plexus (cp) of the dorsal 3^rd^ ventricle. The box indicates the approximated position depicted in panels (**b–e**). For *in vivo* uptake into the brain **(b)** 500 µM of alkyne oleate was continuously applied to the blood circulation of mice for 20 min, or **(c**–**e)** 3 mM alkyne oleate was injected as a single dose into the lateral brain ventricle before incubation for 1 h. Fluorescence microscopy after click-reaction was performed and merged channel overview micrographs of the medial habenula are shown. To identify **(d)** oligodendrocytes and their precursors or **(e)** astrocytes, samples were immuno-probed for Olig2 or GFAP, respectively. Inserts depict close-up images of individual cells with co-localizing signals. Asterisks indicate a blood vessel. Scale bars, (**a**) 200 μm; (**b**–**e**) 50 μm.

The strong labelling in the grey matter region of the medial habenula by the different fatty acids in cultured brain slices (Figs 1c and 3) pointed to a physiological relevance of the local lipid metabolism in this area. To compare the lipid distribution in the medial habenula to that in adjacent brain structures, large overview images of brain slices were recorded (Fig. 4a). These covered a region including the medial and lateral habenular nuclei, fasciculus retroflexus, paraventricular thalamic nucleus, dentate gyrus and dorsal 3^rd^ ventricle. The choroid plexus and the ependymocytes, both directly facing the ventricle, as well as the parenchyma of the medial habenula were labelled by alkyne oleate and all other tested fatty acids (Fig. 4a, compare Fig. 1c). In the habenular parenchyma co-staining of individual astrocytes (Fig. 4a, compare Fig. 3d, right) and oligodendrocytes (Fig. 3b, right) identified by their tdTomato expression was observed. The lipid labelling also included the inter-cell space and cells not marked by the tdTomato reporter (Figs 1c, 3 and 4a). Strikingly, the strong lipid signal observed in the medial habenula did not spread substantially into adjacent brain areas (Fig. 4a), pointing to local peculiarities in lipid transport or metabolism in the confined area of the medial habenula.

To corroborate these findings from the slice cultures and compare lipid targeting to the medial habenula *in situ* and *in vivo*, two sets of *in vivo* experiments in mice were performed. The alkyne fatty acid tracer was administered either via the blood circulatory system (Figs 4b and S3) or by intracerebroventricular injection (Fig. 4c–e). For the former a constant delivery over 20 min time and for the latter a single application followed by 1 h incubation was chosen as experimental time frames. Both settings resulted in substantial lipid accumulation in the medial habenula, but showed differences in the internal signal distribution in this brain area. While the limiting layer of ependymocytes was strongly stained in both *in vivo* settings, only the lipid delivery via the circulatory system resulted in strong staining of the medial habenular parenchyma. Here, also blood vessels became discernable as identified by their morphology (Fig. 4b). In contrast, the lipid signal after intracerebroventricular injection was largely confined to the ependymocyte cell layer and the first 50 μm of the underlying parenchyma (Fig. 4c).

To confirm the involvement of habenular macroglia in local lipid metabolism also *in vivo*, a set of microscopy co-localization experiments with immuno-identification of the lipid-labelled cells was performed. Immuno-labelling of oligodendrocytes and their precursors all positive for the Olig2 marker (Fig. 4d) and of astrocytes expressing the GFAP marker (Fig. 4e) verified a role of these macroglial cell types in local lipid processing.

The *in vivo* distribution of the lipid tracer after delivery via the blood circulatory system was also analysed in other grey matter regions like the thalamus and cortex, as well as in the white matter structures of corpus callosum, fimbria hippocampi and internal capsule (Supplementary Fig. S3). As discerned by its morphology the capillary endothelium was strongly labelled in all investigated brain areas. Otherwise the signal distribution in these areas largely matched that observed in the corresponding *in situ* setup, brain slices incubated with alkyne oleate (Supplementary Fig. S3, compare Figs 1 and 2). Identification of lipid labelled oligodendrocytes and astrocytes by immuno-staining or by employing transgenic fluorescent reporter mice (Supplementary Fig. S3) confirmed the lipid targeting of both macroglial cell types and their involvement in local lipid processing under *in vivo* conditions.

### Oligodendrocytes and astrocytes perform lipid biosynthesis *in vitro*

**Figure 5:**
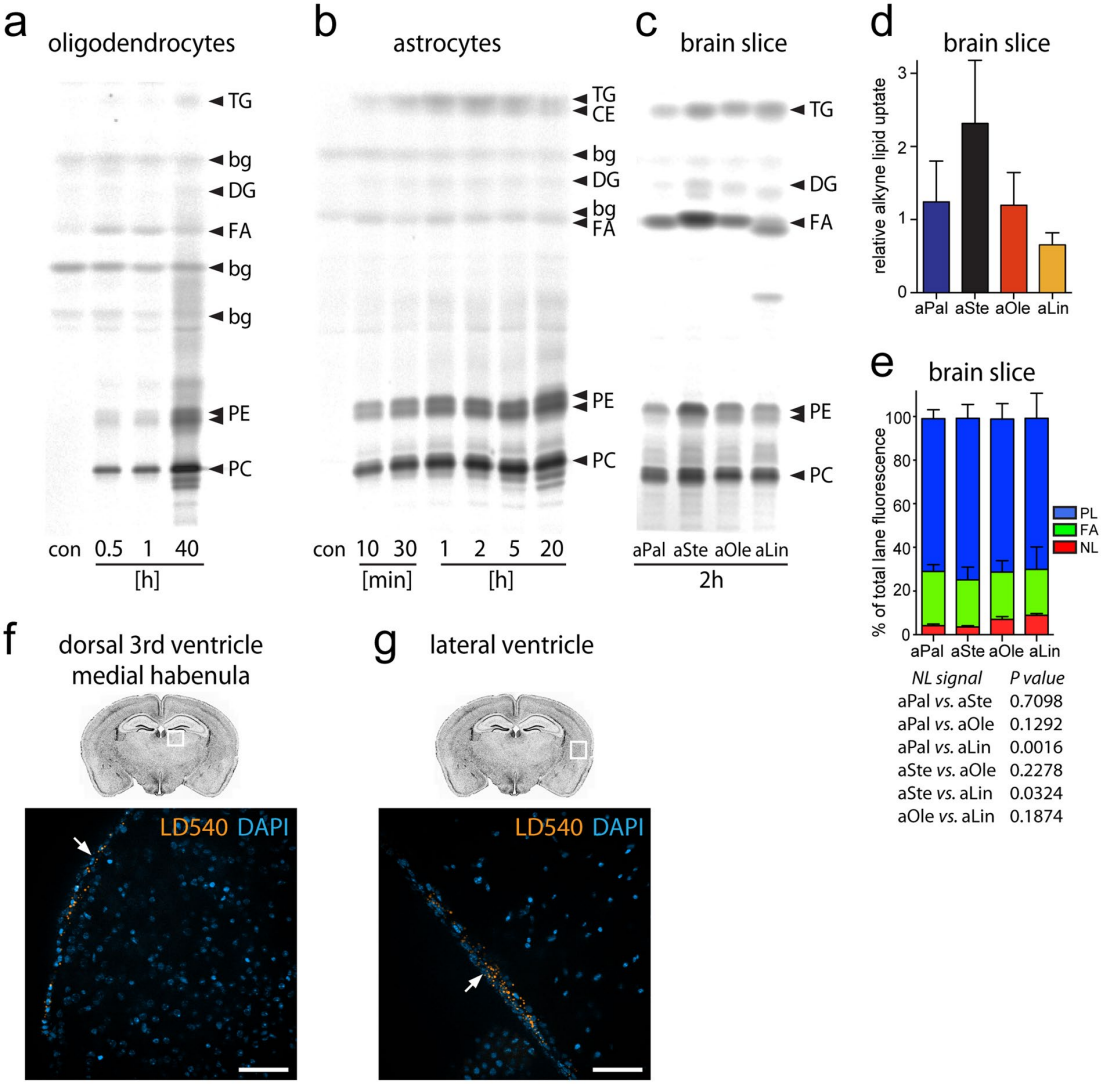
Analysis of lipid metabolites in astrocytes, oligodendrocytes and mouse brain slices. (**a)** Rat oligodendrocytes or **(b)** astrocytes were incubated with alkyne oleate (aOle) or without (con) for the indicated times. Cellular lipid lysates were prepared before click-reaction and TLC analysis. A fluorescence image of the TLC plate was recorded depicting alkyne lipid metabolites, identified by synthetic standards comigrating on the same or on parallel plates. Cropped images are shown here; full-area images of the plates are presented in Supplementary Fig. 4. **(c)** Brain slices of mice were incubated with alkyne palmitate (aPal), alkyne stearate (aStea), aOle, or alkyne linoleate (aLin) for 2 h. After excising the hypothalamus lipid lysates of the remaining slice were prepared analysed. TG: triacylglycerol; CE: cholesterol ester; DG: diacylglycerol; bg: background; FA: free fatty acid; PE: phosphatidylethanolamine; PC: phosphatidylcholine. **(d)** Relative quantification of alkyne lipid uptake into the brain slices ex hypothalamus. Total alkyne lipid signal in the respective lane of panel (c) was normalized to the signal intensity of non-fluorescent lipid content (sum of natural PC, PE, and cholesterol bands) quantified from the same lane after charring of the TLC plate. **(e)** Relative quantification of band intensities from panel (c) as percentage of the total alkyne lipid signal in the respective lane. NL, neutral lipid (TG + CE); PL, phospholipid (PC + PE). For (**d**,**e**) mean values and s.e.m. are plotted (n = 3). Details on the statistical analysis of labeled NL content from a one-way ANOVA are shown in the lower subpanel. **(f**,**g)** Localization of NL in lipid droplets. Mouse brain slices were cultured for 2 h before fixation and probing for lipid droplets (LD). Fluorescence micrographs show lipid droplets (LDs; orange) and nuclei stained by DAPI (blue) in merged channels overview images. Arrows point to the ependymocyte cell layer of the (**f**) dorsal 3^rd^ and (**g**) lateral ventricle, containing the vast majority of all detected LDs. All images were recorded with equal settings and all colour images are shown at equal intensity levels. Scale bar: 50 µm.

To investigate the capability of oligodendrocytes and astrocytes for lipid biosynthesis more detailed, individual primary cultures of both macroglia were incubated with alkyne oleate for various times. The cellular alkyne lipid metabolites of both cell types were separated by thin-layer chromatography (TLC) (Fig. 5a,b). Analysis showed that both macroglia readily take up and use the fatty acid for biosynthesis of a variety of membrane lipids. Already at the earliest time point investigated (30 min), the cells contained labelled phosphatidylcholine and –ethanolamine, the prevalent membrane phospholipids. Astrocytes also rapidly produced substantial amounts of the neutral lipids cholesterol ester, di- and triacylglycerol, whereas in oligodendrocytes only minor amounts of these storage lipids were detectable, even after very long incubation times.

Next the lipid biosynthesis in cultured brain slices was studied (Fig. 5c–e). For this, slices were incubated with different fatty acids for 2 h. As the lipid metabolism in the hypothalamus likely differs from that in other brain areas^15^, the hypothalamic portion was excised from slices and analysed separately^16^. While all fatty acids were readily taken up from medium (Fig. 5d) and became incorporated into the different lipid classes (Fig. 5e), the samples incubated with the unsaturated oleate and linoleate showed slightly higher relative amounts of labelled neutral lipids than the samples treated with saturated fatty acids. As neutral lipids are commonly stored in a dedicated cellular organelle, the lipid droplet (LD)^17^, a microscopy analysis of the LD distribution in the slices was performed. The vast majority of LDs was detected in the ventricle regions, where the ependymocytes contained large amounts of LDs of different size (Fig. 5f,g). Only negligible amounts were detectable in the parenchyma of the medial habenula or elsewhere.

## Discussion

The present study aimed at investigating the lipid metabolism in selected murine grey and white matter structures in order to explore the macroglial involvement in local lipid processing. For this, we have employed the *in situ* system of acute brain slice cultures and state-of-the-art alkyne lipid tracers that exceptionally match natural lipids in structure and biological behavior^18, 19^. This technique allows for parallel lipid tracking by both fluorescence microscopy in order to determine lipid distribution, and TLC analysis to identify and quantify lipid metabolites^20, 21^.

We have compared four alkyne fatty acids from the saturated and unsaturated families. In white matter all fatty acids were similarly targeting cells many of which we identified as oligodendrocytes and astrocytes by microscopy co-labelling experiments. Noteworthy, also other unidentified cells, potentially neurons or microglia, became lipid-labelled but these were not investigated further in this study.

Oligodendrocytes and astrocytes represent the two dominant glia types in the mammalian brain^22^ and white matter contains a significantly higher glia-to-neuron ratio than grey matter^23^. Jointly both macroglial cell types provide the necessary lipids for incorporation into myelin^24, 25^. Among the fatty acids occurring in myelin lipids, about 80% have a chain length of 18 carbon atoms or less and 6% are PUFAs^13^, albeit other numbers have been obtained (60% and 20%, respectively)^25^. Accordingly, when comparing palmitate, stearate, oleate and linoleate, we did not detect a chain-length or saturation dependent difference in fatty acid targeting to white matter arguing against a biased uptake.

For grey matter a less uniform picture emerged. We found indications for regional differences between various grey matter structures. While all fatty acids strongly labelled the medial habenula, saturated fatty acids only moderately labelled the thalamus and cortex. However, the unsaturated alkyne oleate and the PUFA-tracer, alkyne linoleate, exhibited increased staining intensities also here. In agreement with this observation, grey matter is known to be enriched in PUFA-containing phospholipids compared to white matter^11, 13^. As in white, also in grey matter we identified the oligodendrocytes and astrocytes as targets for the lipids both in brain slices and in animal experiments. Hence, our data support the notion that these glia cells are profoundly engaged in cerebral lipid metabolism also under *in vivo* conditions. Unlike in slices, here a fully functional blood-brain barrier operates and the macroglia are downstream of this barrier. Therefore, even as we supplied the tracer as free fatty acid it may reach the parenchymal macroglia in other forms, potentially carried by cerebral lipoproteins.

Using primary cultures of cortical oligodendrocytes and astrocytes we confirmed for both macroglial cell types individually their capacity for lipid biosynthesis. Already at the early time points after feeding (10–30 min) a similar profile of phospholipid metabolites was found. Both cell types have been shown to synthesize phospholipids *de novo* from acetate^26^ or fatty acids^27^. Noteworthy, compared to oligodendrocytes the astrocytes showed a higher relative content of the labelled neutral lipid triacylglycerol throughout the course of the experiment. This pointed to the possibility that within the brain, astrocytes are a main producer of neutral lipid stored in LDs^17^. However, when we investigated the distribution of LDs in brain slices, the majority of the LD signal was confined to the ependymocytes at the ventricle walls, and astroglial LDs could not be detected either in grey or in white matter. This finding matches our analysis of the hypothalamic LD distribution^16^. In conclusion, our TLC data illustrates the fact that astrocytes possess the enzymatic machinery needed for neutral lipid synthesis, but in tissue do not produce these lipids for storage under physiological conditions. While the brain generally contains little neutral lipid^28–30^, our findings indicate that the ependymocyte layer accommodates substantial amounts of it. As ependymocytes are lining the ventricular system filled with cerebrospinal fluid, they are in a prime position for exchange processes between this fluid and the brain parenchyma. In line with this, we observed a strong lipid staining in ependymocytes, indicative for a high fatty acid uptake and (neutral) lipid biosynthesis activity.

When available to cells, fatty acids might serve as building blocks for the synthesis of other lipids or serve a catabolic function through β-oxidation. It is important to keep in mind that alkyne tracers do not allow for following β-oxidation as this catabolic process likely eliminates the tracer from the cells. Accordingly, the end products of β-oxidation would not be detectable either by microscopy or by TLC analysis. Hence, this study did not investigate the catabolism of the fatty acids.

The medial habenula, a grey matter structure analysed in this study, showed intense lipid staining, indicating a highly active local lipid metabolism. The medial and lateral parts of the habenula form a central structure connecting forebrain to midbrain regions. The habenula integrates cognitive with emotional and sensory processing, encoding both rewarding and aversive aspects of external stimuli^31^. The habenula regulates monoaminergic systems and contributes to learning and memory^32^.

We found that the medial but much less the lateral habenula was targeted by fatty acids. Unlike the hypothalamus^16^, another grey matter region in contact with the ventricular system, the medial habenula did not show discrimination in uptake of saturated *vs*. unsaturated fatty acids. We validated our findings from the *in situ* setup using two *in vivo* models. Largely they confirmed our results from the slice cultures and the small differences occurring, likely are of technical origin. Lipid application via the circulation stained the habenular parenchyma more efficiently than delivery by intracerebroventricular injection. In the latter approach the amount of fatty acid tracer that can be administered is limited by the injection volume (3 µL) and the solubility of the lipid tracer. Hence, we potentially did not deliver sufficient amounts of fatty acid to the ventricular system that upon dilution with the cerebrospinal fluid would allow tracer penetration deeper than the observed 50 µm and warrant staining of the habenular parenchyma. Also possible, the accessibility to the parenchyma for fatty acids transported solely by the cerebrospinal fluid is lower compared to that for circulation-derived lipids.

While our data demonstrate a profound lipid uptake to the medial habenula, the physiological relevance of this observation is unclear. One might speculate about an influence of lipids on the local processing of various external stimuli. The local expression of cannabinoid type 1 receptor (CB1R) in the medial but not lateral habenula may hint in this direction, as CB1R provides a link between lipid signals and neuronal processing^33^. However, to substantiate this speculation a possible connection clearly has to be investigated further.

In summary, the data presented here reinforce the role of two macroglial cell types, oligodendrocytes and astrocytes found in grey and white matter, as components of the brain lipid metabolism machinery. Our findings connect to the established function of oligodendrocytes in myelin production and the notion that astrocytes are a main producer of lipids in the brain, providing these lipids also to other cerebral cells^13, 24–27, 34^. The involvement of both glia cell types in fatty acid uptake and lipid metabolism, investigated here *in vitro*, *in situ* and *in vivo*, deepens our knowledge on glial activities.

## Materials and Methods

### Reagents

Alkyne fatty acids were synthesized as described: Alkyne linoleate^35^, alkyne palmitate and alkyne oleate^18^. Alkyne stearate (17-octadecynoic acid), azide-PEG3-biotin conjugate, [acetonitrile]_4_CuBF_4_ and DAPI were obtained from Sigma Aldrich, lipid free BSA from Applichem. LD540 was described before^36^. Antibodies against GFAP (Synaptic systems, 173004) and Olig2 (Millipore, AB9610) were combined with secondary antibodies from Invitrogen and fluorescent streptavidine-Alexa 488 conjugate from Dianova. Fluorescent mounting medium was from Dako.

### Primary cell culture and lipid supplements

Primary rat OPCs and astrocytes were isolated from Wistar rat pups at P0-P2 by a differential detachment method applying slight modifications to an established protocol^37^. Briefly, cerebra were incubated with trypsin for 15 min before deactivation of trypsin and mechanic dissociation. The clump-free cell suspension was plated into 75-cm^2^ culture flasks with astrocyte growth medium (DMEM supplemented with 10% (v/v) heat-inactivated foetal calf serum (FCS), penicillin (100 units/ml), streptomycin (0.1 mg/ml) and Mito Serum Extender (Becton-Dickinson)) with the medium exchanged after 2 days. After 10–12 days, mixed cultures were shaken (260 rpm) for 14 h to detach OPCs and microglia from astrocytes. Astrocytes were cultured for additional 2–6 days before use. OPCs were further enriched by plating the detached cell mix onto uncoated Petri dishes (Corning) for 30 min. Then, the non-adherent OPCs were seeded into poly-L-ornithine-coated plates and maintained in proliferating Neurobasal medium supplemented with 2% (v/v) B27, 2 mM GlutaMAX, 100 units/ml penicillin, 0.1 mg/ml streptomycin, 20 ng/ml PDGF-AA, and 20 ng/ml basic FGF for 3–4 days (37 °C, 5% CO_2_), changing the medium every second day. Thereafter, medium was switched to growth factor-free Neurobasal medium supplemented with 20 ng/ml triiodothyronine (T3) for 7 days. For lipid incubation 50 µM of the alkyne fatty acid was added to the respective growth medium which in case of oligodendrocytes was supplemented with 1% lipid free BSA.

### Animals

Wistar rats were maintained in the animal facility of the University Hospital Bonn. Wild-type C57BL/6NCrl mice (Charles River) were maintained in the animal facilities of the University of Barcelona or the University of Bonn and generally fed a breeding and maintenance diet (LASQCdiet^®^ Rod16, LASvendi) *ad libitum*. For the canulation experiments, male mice were used at the age of 8 weeks. For other experiments, male wild-type animals were used at the age of 18 weeks after feeding a normal control diet (10 kcal% fat; C1090-10, Altromin Spezialfutter) *ad libitum* for 10 weeks. Transgenic mice were held at the animal facility of the CIPMM and generally fed a breeding diet (V1125, Ssniff) *ad libitum*. Mice expressing the inducible Cre DNA recombinase variant CreERT2 under control of the murine PLP promoter (PLP-CreERT2)^38^ or knocked into the GLAST locus (Slc1a3^tm1(Cre/ERT2)Mgoe^, ref. 39) were crossbred with reporter mice expressing tdTomato after tamoxifen induced recombination (Gt(ROSA)26Sor^tm27.1(CAG-COP4*H134R/tdTomato)Hze^, ref. 40). Reporter mice of both genders were used. All animals were held at a twelve-hour light/dark cycle.

This study was carried out in strict accordance with the European, Spanish and German guidelines for the welfare of experimental animals. Animal experiments were approved by the University of Barcelona local ethics committee in agreement with the Spanish legislation (BOE 32/2007) and European Directive 2010/63/EU for ethical management of animals, the North-Rhine-Westphalia and Saarland state’s “Landesamt für Natur, Umwelt und Verbraucherschutz” and “Landesamt für Gesundheit und Verbraucherschutz” animal license numbers: 425/16, 84-02.04.2015.A381 and 65/2013, respectively.

### Tamoxifen injections

To induce DNA recombination in both inducible CreERT2 mouse lines, tamoxifen (Sigma-Aldrich), dissolved in corn oil (10 mg/ml, Sigma-Aldrich) was intraperitoneally injected into 7 till 10-weeks-old mice for 3 consecutive days (100 mg/kg body weight). Mice were analysed 8 till 12 days after the first injection^41^.

### *In vivo* application of alkyne lipids

For blood vessel administration, mice were transcardially perfused sequentially with 500 µM alkyne oleate in oxygenated Ringer solution (0.7 mM Na_2_HPO_4_; 1.3 mM NaH_2_PO_4_; 15 mM Na_2_CO_3_; 10 mM glucose; 0.49 mM MgCl_2_; 4.56 KCl; 120 mM NaCl; 25 g/L lipid free BSA) and 4% PFA in PBS at a flow rate of 18 mL/min for 20 min each. For intracerebroventricular administration a protocol previously described^42^ was slightly modified: Briefly, brain infusion cannulae were stereotaxically placed in the lateral ventricle (0.58 mm posterior to bregma; 1 mm lateral to the midsagittal suture and to a depth of 2.2 mm). Cannula placements were verified by assessing a rapid drinking response to angiotensin II. After 4 days, a 3 µL bolus injection of 3 mM alkyne oleate in Ringer solution was administered. After 1 h mice were transcardially perfused as described below.

### Perfusion and immunohistochemistry (IHC)

For IHC analyses mice were transcardially perfused sequentially with PBS and 4% PFA in PBS at a flow rate of 18 mL/min. Brains were incubated in fixative for 2 d. Slices (30 µm thickness, vibratome) were fixed applying 4% PFA in PBS for 10 min before washing with PBS. Blocking buffer (1% BSA, 0.1% Triton X-100 in PBS) was added for 1 h before incubation with primary antibody in blocking buffer at 4 °C overnight. PBS was used for washing, incubation with secondary antibody, and DAPI staining before mounting.

### Microtomy, slice culture and lipid supplement

Acute brain slices were prepared using a protocol previously described^15, 43^ with slight modifications. Dissected mouse brains were directly embedded in artificial cerebrospinal fluid (ACSF, 6.25 mM NaHCO_3_, 31 mM NaCl, 0.65 mM KCl, 0.25 mM MgCl_2_, 0.5 mM CaCl_2_, 0.35 NaH_2_PO_4_, 6.25 mM glucose) containing 4% agarose before slicing (250 µm thickness, vibratome) in ice cold oxygenated ACSF. The time between sacrificing the animal and begin of slice cultivation was routinely less than 1 h. Slices (bregma level −1.3 to −2.0) were cultured at 37 °C in medium A (50% glucose-free DMEM, 25% horse serum (Gibco), 25% Hanks Balanced Salt Solution, 25 mM glucose, penicillin/streptomycin) for 2 h while constantly oxygenating using carbogen bubbling. For lipid incubation 50 µM from an ethanol stock solution of the alkyne fatty acid was added to medium A.

### Sample collection for lipid extraction and thin-layer chromatography (TLC) analysis

Slices or cells cultured as above were washed with PBS containing 1% lipid free BSA. From slices the hypothalamus was excised and only the remainder homogenized in 1 mL methanol/chloroform (1/1). Phase separation was induced by adding 800 µL water and 200 µL chloroform. The organic phase was retrieved, evaporated and redissolved in 10 µL chloroform.

Cells were scraped in 1 mL ammonium acetate and added to 5 mL methanol/chloroform (3/1) before centrifugation and retrieval of the supernatant. Phase separation was induced by adding 5 mL water and 2 mL chloroform. The organic phase was retrieved, evaporated and redissolved in 10 µL chloroform.

### Alkyne lipid detection

In general, copper(I)-catalysed azide-alkyne cycloaddition, CuAAC^44, 45^, frequently termed click chemistry^46^, was employed to detect alkyne lipids.

### Click reaction for lipid analysis by thin-layer chromatography (TLC), imaging of TLC plates, signal quantification

Cellular alkyne lipid metabolites were analysed by reacting the lipid extracts with 3-azido-7-hydroxycoumarin before TLC analysis and fluorescent plate imaging as described previously^18^. Images (512 × 512 px) were acquired with a Rolera MGI plus EMCCD camera (Decon Science Tec), equipped with 494/20 and 572/28 bandpass emission filters. To reduce some background bands with broad fluorescence emission the picture obtained in the 572 nm (noise) channel was subtracted from that obtained in the 494 nm (signal) channel. Finally, the natural lipids were quantified by charring the same TLC plates using 20% sulphuric acid and densitometry analysis. For signal quantification the Gel-pro analyser (Media Cybernetics) and Fiji^47^ software bundles were used.

### Click reaction, lipid detection by fluorescence microscopy

Lipid localization was analysed by fluorescence microscopy using a biotinylated azide reporter and a protocol previously described^16, 20^. In brief, slices were fixed applying 4% PFA in 100 mM phosphate buffer (pH 7.5) at 4 °C for 16 h, before washing sequentially with PBS, 155 mM ammonium acetate and twice with buffer A (50 mM HEPES/KOH, pH 7.5). Mild permeabilization was performed using 0.1% saponin in buffer A for 30 min followed by the click reaction. For the latter, 50 µM biotinylated azide reporter in 400 µL prewarmed buffer A was added to the samples. The click reaction was initiated by addition of 2 mM [acetonitrile]_4_CuBF_4_ in acetonitrile (final 2% acetonitrile) and performed at 43 °C for 60 min without agitation. Samples were extensively washed using buffer A and afterwards PBS. Slices of fluorescent reporter mice were additionally washed with 100 mM EDTA in water. Finally, slices were incubated with 300 µL of (2 mg/mL) fluorescent streptavidine-Alexa 488 conjugate and if applying with DAPI before mounting. All microscopy experiments were performed as full biological and technical replicates (n ≥ 2). Negative control samples lacked alkyne lipid incubation but were otherwise equally processed.

For staining of lipid droplets, fixed slices were washed twice with PBS and incubated sequentially with LD540^36^ and DAPI before mounting.

### Microscopy

Epifluorescence microscopy was performed using a Zeiss Observer.Z1 microscope (Carl Zeiss) equipped with a Fluar 40× (1.30 NA) objective and a Photometrics Coolsnap K4 camera. Optical sectioning was performed using the apotome mode. The light source was a Polychrome V 150 W xenon lamp (Till Photonics). All images were processed employing the ZEN (Carl Zeiss) and Adobe Photoshop 6.0 (Adobe) software packages. If applying, projections of z-stacks and tilted views thereof were calculated by summarizing corresponding pixel values using ZEN or Fiji^47^ software.

To illustrate the white and grey matter regions covered in this study a full brain slice overview image depicting DAPI-stained nuclei was generated with an Axio Scan.Z1 (Zeiss, Jena, Germany) using an HBO lamp (HXP 120 V, LEJ, Jena, Germany) for excitation and a Plan-Apochromat 10× (0.45 NA) objective for the course focus map and a Plan-Apochromat 20× (0.8 NA) objective for the fine focus map with appropriate emission and excitation filters. Images were recorded in 8 μm thick stacks and a variance intensity projection was prepared for representation.

### Semi-quantification of microscopy images

For the semi-quantitative analysis of alkyne lipid localization samples were processed identically on different days. For recording of all micrographs identical acquisition settings were applied. All semi-quantification was performed using Fiji^47^ software and the following procedures. A ‘region of interest’ (ROI) that covered the indicated brain region was specified for each micrograph individually. From the ROI the ‘mean signal intensity’ (lipid fluorescence) and the area were determined and the ratio ‘mean signal intensity per pixel’ calculated. For background correction corresponding values from micrographs of control samples that lacked alkyne lipid during incubation were subtracted to yield the relative ‘mean signal intensity per pixel’. Average values ± SEM, n = 1 to 6 are presented in Supplementary Table S1.

### Data availability

The datasets generated during and/or analysed during the current study are available from the corresponding author on reasonable request.

## Electronic supplementary material

Supplementary Material
