# Supplementary material for: Astrocytes and oligodendrocytes in grey and white matter regions of the brain metabolize fatty acids
Source: Sci Rep. 2017 Sep 7;7:10779. doi: 10.1038/s41598-017-11103-5 (PMC5589817; doi:10.1038/s41598-017-11103-5)
Supplement: Supplementary file 1 — Supplementary Material [file 41598_2017_11103_MOESM1_ESM.pdf]

Supplementary Figures S1-S4 and Supplementary Table S1 to

Astrocytes and oligodendrocytes in grey and white matter regions of the brain metabolize fatty acids

by

Kristina Hofmann, Rosalia Rodriguez-Rodriguez, Anne Gaebler, Núria Casals, Anja Scheller, Lars Kuerschner

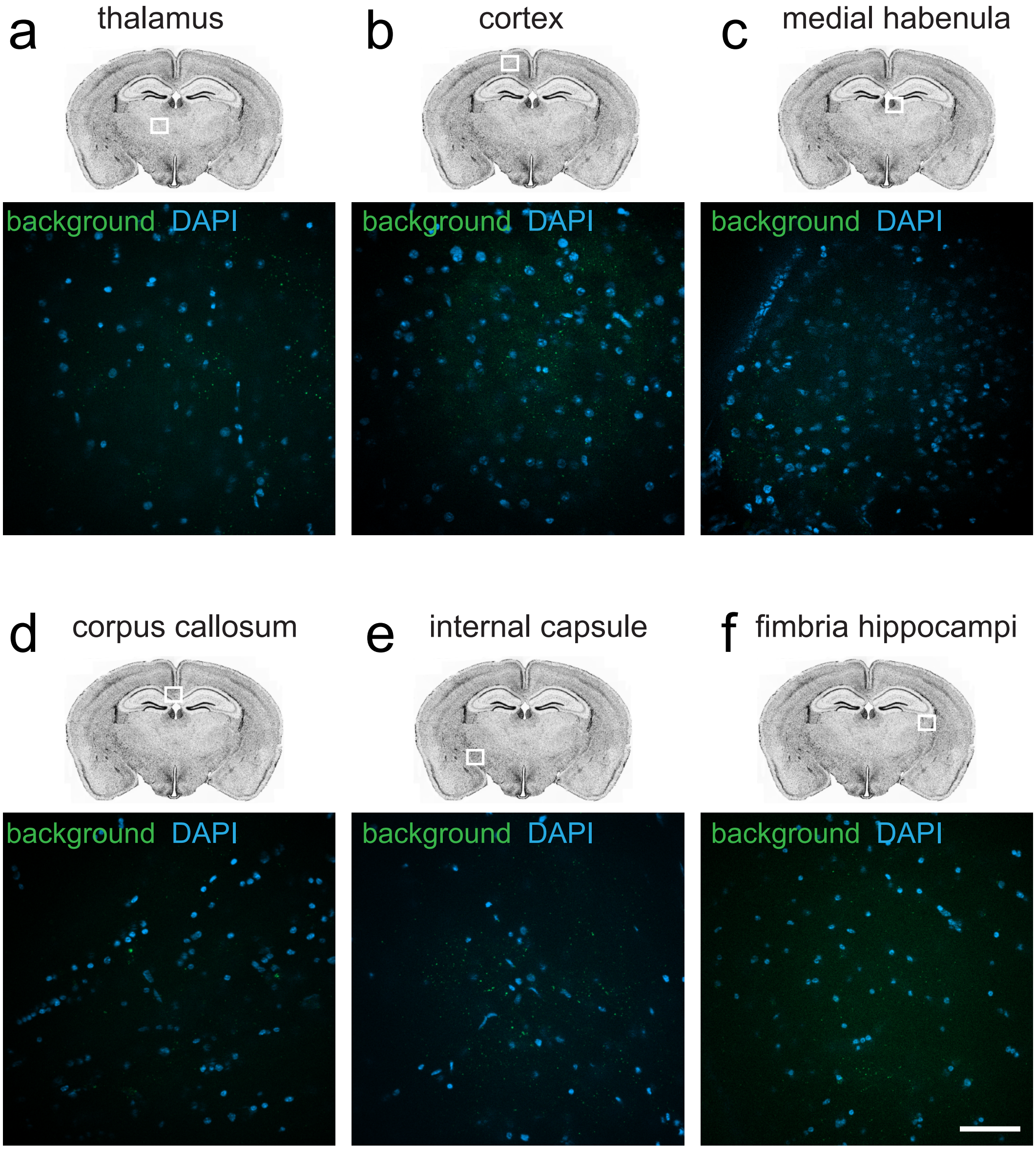

Supplementary Figure S1: Samples lacking alkyne lipid tracers during cultivation show minor background lipid signal. As negative controls brain slice cultures of mice were incubated in the absence of alkyne fatty acid for 2 h. Fluorescence microscopy after click-reaction was performed showing the background noise in the lipid channel (green) and nuclei (DAPI). Merged channel overview micrographs were recorded and are presented with settings matching the Figs. 1-2 of the main text. Scale bar: 50 μm.

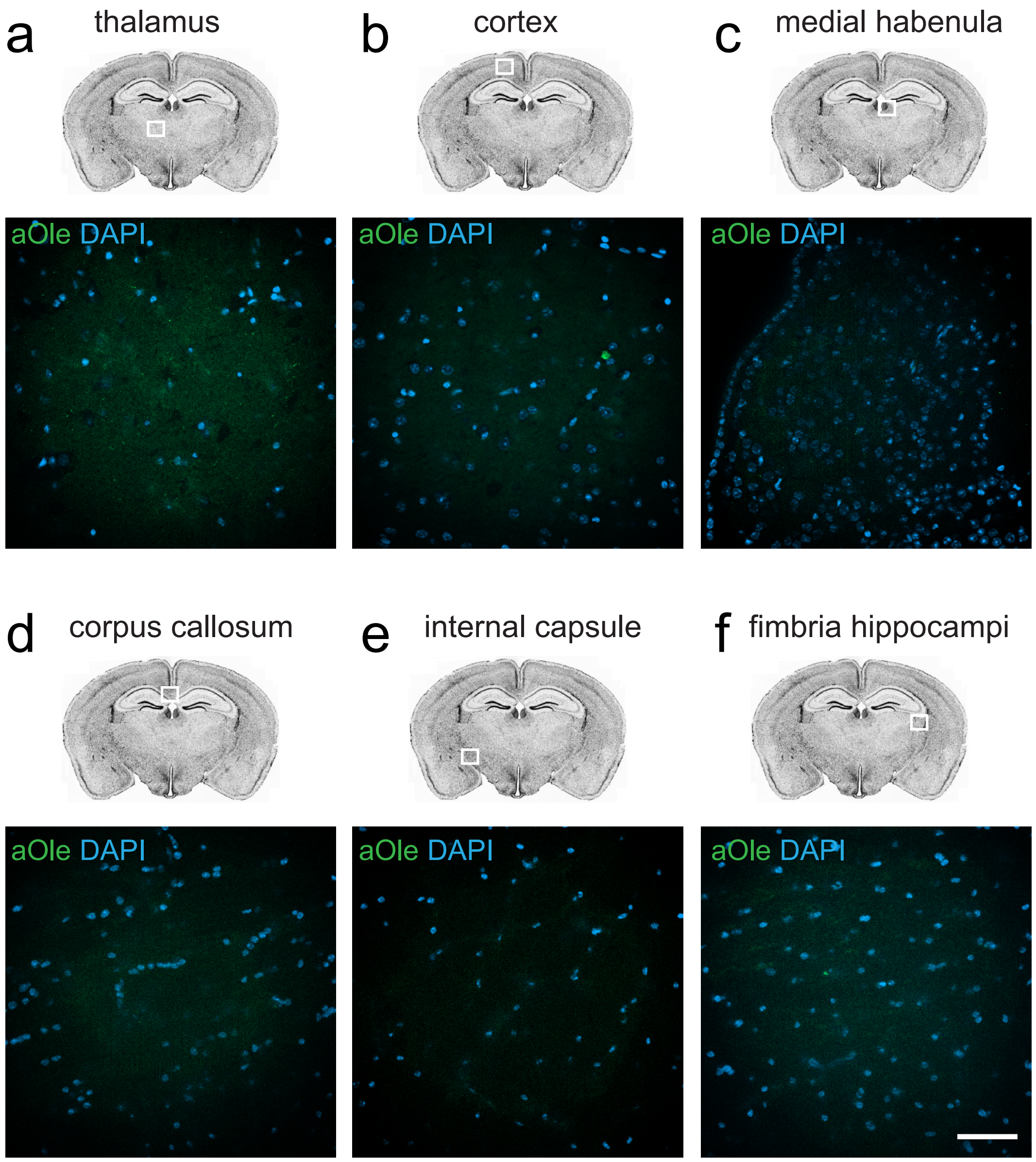

Supplementary Figure S2: Samples prefixed before incubation with alkyne lipid tracer show minor background lipid signal. As negative controls brain slice cultures of mice were first pre-fixed using aldehyde and the dead slice incubated with 50 μM of alkyne oleate (aOle) for 2 h. Fluorescence microscopy after click-reaction was performed showing background noise in the lipid channel (green) and nuclei (DAPI). Merged channel overview micrographs were recorded and are presented with settings matching the Figs. 1-2. Scale bar: 50 μm.

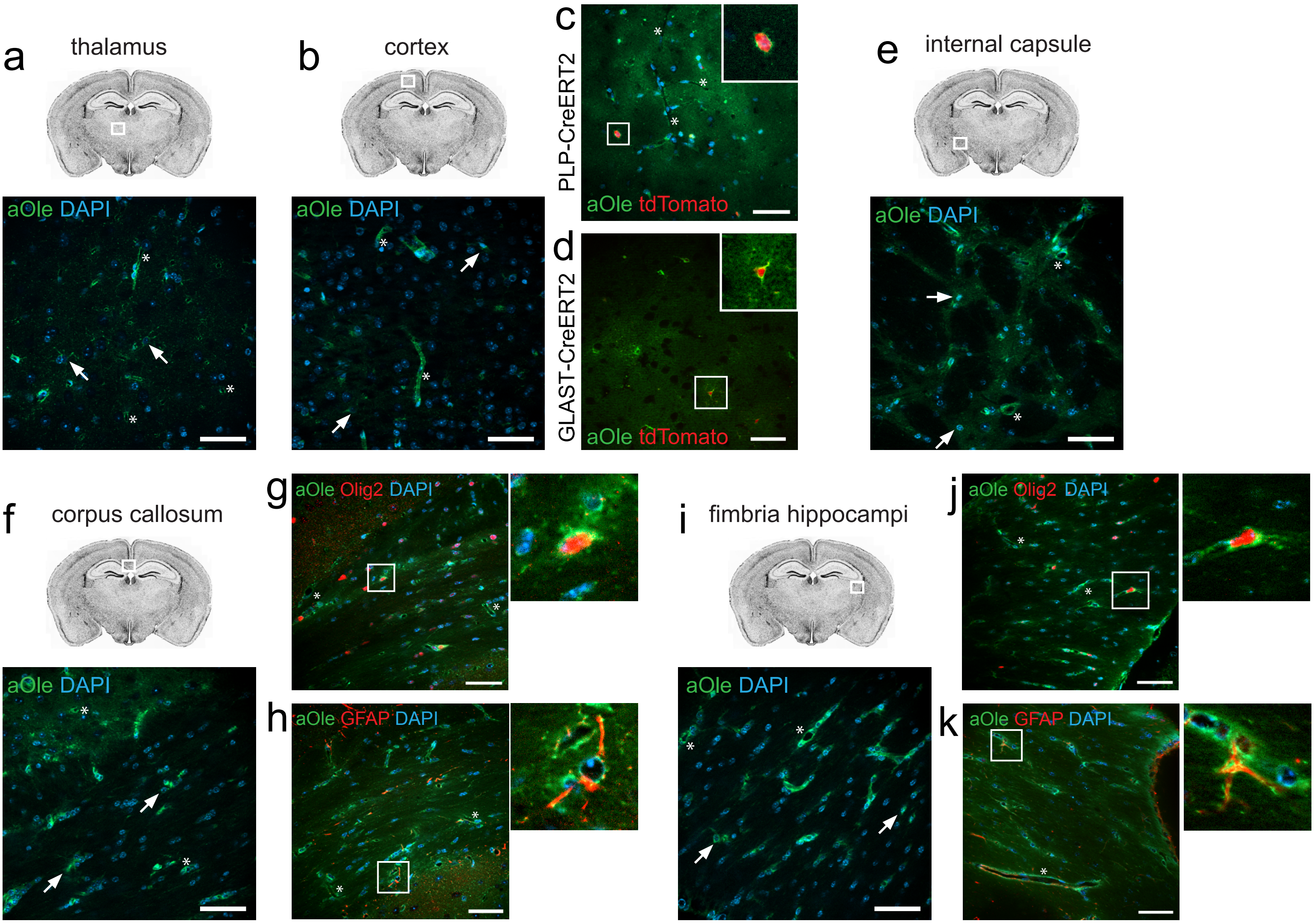

Supplementary Figure S3: The alkyne lipid uptake to grey and white matter in vivo corresponds to the in situ observations. For in vivo uptake into the brain 500 μM of alkyne oleate was continuously applied to the blood circulation of wildtype mice (a,b,e-k) or transgenic mice expressing the red-fluorescent tdTomato-reporter after tamoxifen-induced recombination in (c) oligodendrocytes (PLP-CreERT2), or (d) astrocytes (GLAST-CreERT2) for 20 min. Fluorescence microscopy after click-reaction was performed and merged channel overview micrographs of (a) thalamus, (b-d) cortex, (e) internal capsule, (f-h) corpus callosum, and (i-k) fimbria hippocampi are shown. Alkyne lipid (green), nuclei stained by DAPI (blue) and marker proteins (red) are depicted. To identify (g,j) oligodendrocytes and their precursors or (h,k) astrocytes, samples were immuno-probed for Olig2 or GFAP, respectively. Inserts depict close-up images of individual cells with co-localizing signals. Asterisks indicate a blood vessel; arrows point to individual cells. Scale bars, 50 µm.

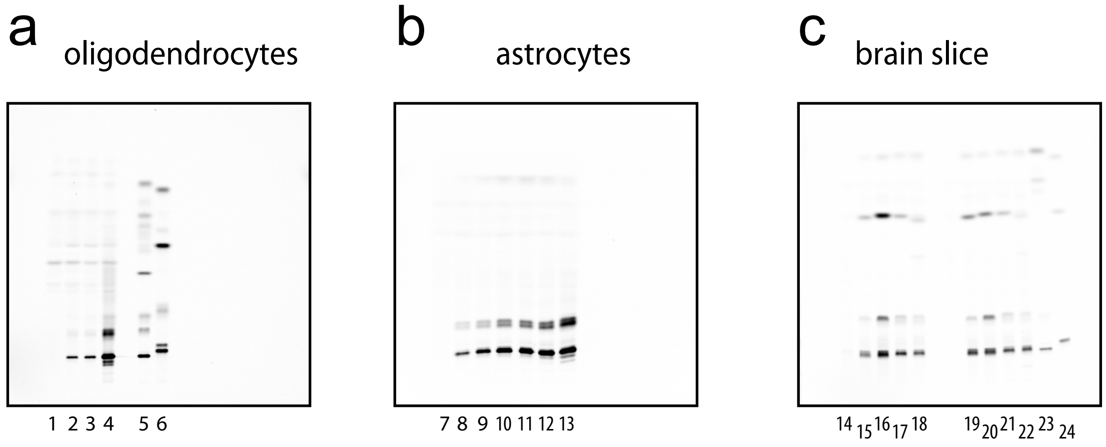

Supplementary Figure S4: Full-area images of the TLC plates presented in Fig. 5 (a-c) of the main text. The recorded images (camera chip size 512x512 px) covering the full area of the TLC plate (20x20 cm) were cropped to the boundaries of the plate and are depicted at a resolution of 300 dpi. Lanes 1-4 are shown in Fig. 5 (a), lanes 7-13 are shown in Fig. 5 (b), and lanes 15-18 are shown in Fig. 5 (c). Lanes 5,6,14,19-24 are not part of Fig. 5. The standards were applied in lanes 5,6 and 23,24. The set of the synthetic lipid standards distributed over both lanes included PC, PE, FA, DG, CE, TG that were characterized individually before (ref.18, main text). PC: phosphatidylcholine; PE: phosphatidylethanolamine; FA: free fatty acid; DG: diacylglycerol; CE: cholesterol ester; TG: triacylglycerol.

Supplementary Table S1. Semi-quantification of the relative alkyne lipid fluorescence signal in various grey and white matter regions.

	mean signal intensity per pixel [a.u.]			
	aPal	aStea	aOle	aLin
thalamus	103 ± 32	87 ± 8	277 ± 69	261 ± 24
cortex	129 ± 29	132 ± 76	481 ± 155	415

Brain slice cultures of mice were incubated with 50 μM of either the saturated fatty acids alkyne palmitate (aPal) or alkyne stearate (aStea), or the unsaturated fatty acids alkyne oleate (aOle) or alkyne linoleate (aLin) for 2 h. Control samples lacked alkyne lipid during incubation. Fluorescence microscopy after click-reaction was performed. The lipid fluorescence (mean signal intensity) from micrographs was determined using Fiji software. Analysis was restricted to 'regions of interest' (ROI) that covered the indicated brain region. Background correction using the signal value in the corresponding control sample was employed. The relative 'mean signal intensity per pixel' (*STDEV, i.e. standard deviation of the mean)* ± SEM (*standard error of the mean, i.e. standard deviation of the mean divided by the square root of n)*, n = 1 to 6 are shown.
